# Epigenomic drivers of immune dysfunction in aging

**DOI:** 10.1111/acel.12878

**Published:** 2018-11-28

**Authors:** Christine R. Keenan, Rhys S. Allan

**Affiliations:** ^1^ The Walter and Eliza Hall Institute of Medical Research Parkville Victoria Australia; ^2^ Department of Medical Biology The University of Melbourne Parkville Victoria Australia

**Keywords:** chromatin, epigenetics, histone modifications, immune aging, immunity, progeria

## Abstract

Aging inevitably leads to reduced immune function, leaving the elderly more susceptible to infections, less able to respond to pathogen challenges, and less responsive to preventative vaccinations. No cell type is exempt from the ravages of age, and extensive studies have found age‐related alterations in the frequencies and functions of both stem and progenitor cells, as well as effector cells of both the innate and adaptive immune systems. The intrinsic functional reduction in immune competence is also associated with low‐grade chronic inflammation, termed “inflamm‐aging,” which further perpetuates immune dysfunction. While many of these age‐related cellular changes are well characterized, understanding the molecular changes that underpin the functional decline has proven more difficult. Changes in chromatin are increasingly appreciated as a causative mechanism of cellular and organismal aging across species. These changes include increased genomic instability through loss of heterochromatin and increased DNA damage, telomere attrition, and epigenetic alterations. In this review, we discuss the connections between chromatin, immunocompetence, and the loss of function associated with mammalian immune aging. Through understanding the molecular events which underpin the phenotypic changes observed in the aged immune system, it is hoped that the aged immune system can be restored to provide youthful immunity once more.

## 1 INTRODUCTION

Old age is associated with reduced immune function that ultimately leads to elderly individuals becoming less responsive to vaccination and more susceptible to a range of infections (Dorshkind, Montecino‐Rodriguez, & Signer, 2009; Kline & Bowdish, 2016). Extensive studies have found a myriad of changes in cellular phenotypes and functions across almost all compartments of the immune system. However, elucidating why these changes occur has proven a much more difficult prospect.

Key molecular changes underpin the time‐related functional decline across different systems and different organisms. These molecular changes have recently been rationalized to become the four primary “hallmarks of aging”: genomic instability, telomere attrition, epigenetic alterations, and loss of proteostasis (Lopez‐Otin, Blasco, Partridge, Serrano, & Kroemer, 2013). Importantly, three of these four primary hallmarks involve changes to chromatin, highlighting the importance of chromatin state for function and health.

In this review, we discuss the evidence that particular changes to chromatin cause the loss of function seen in the aged immune system. Increased understanding of the connections between phenotypic changes and the underlying molecular events is hoped to reveal key mediators which can be therapeutically targeted to restore immunity (and “youth”) in old age.

## 2 THE AGING IMMUNE SYSTEM

**Figure 1:**
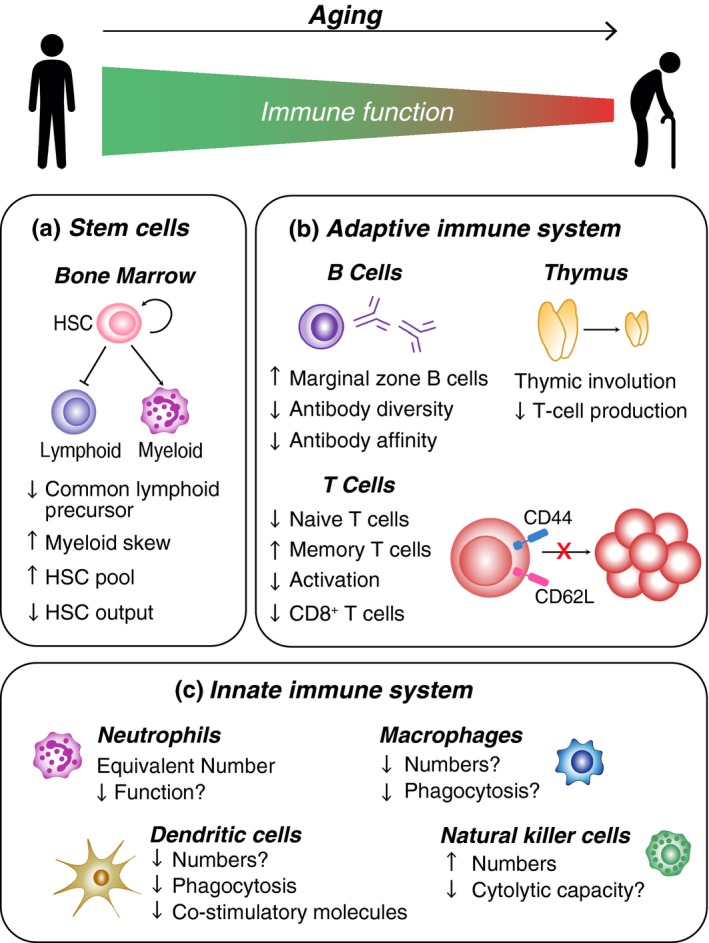
Dysfunction of the aged immune system. Changes in cellular frequency and function compromise the immune health of elderly individuals. These changes are most clearly delineated in the hematopoietic stem cell (HSC) compartment (a), and the adaptive immune system (b), but changes are also seen in the innate compartment (c)

Changes in both the innate and adaptive arms of the immune system have been documented in aging, although the changes in the adaptive immune system are more well‐defined (Figure 1). These changes have been recently reviewed elsewhere (Goronzy & Weyand, 2017; Michel, Griffin, & Vallejo, 2016; Pereira & Akbar, 2016; Pinti et al., 2016; Yanes, Gustafson, Weyand, & Goronzy, 2017). However, key changes in immune cell frequency and function will be described here for completeness. The intrinsic functional reduction in immune competence is associated with low‐grade chronic inflammation, termed “inflamm‐aging,” recently reviewed in Frasca and Blomberg (2016), characterized by high levels of circulating cytokines and latent viral infections. This inflammatory state then further perpetuates the age‐related immune dysfunction.

### 2.1 Hematopoietic stem cells (HSCs)

Hematopoietic stem cells (HSCs) are responsible for the maintenance and replenishment of immune and hematopoietic cells throughout life. HSC numbers actually increase with age, both in mice and humans, but these accumulated cells show reduced reconstitution potential (Morrison, Wandycz, Akashi, Globerson, & Weissman, 1996; Ogawa, Kitagawa, & Hirokawa, 2000; Pang et al., 2011; Rossi et al., 2005). Stem cell diversity also appears to reduce with age with an exponential increase in the occurrence of clonal hematopoiesis, where one mutant HSC produces an overwhelming proportion of mature blood cells, especially after the age of 45–60 in humans (Genovese et al., 2014; Jaiswal et al., 2014; McKerrell & Vassiliou, 2015). Moreover, the output of these aging HSCs is also biased toward the myeloid lineage at the expense of lymphoid cells (such as T and B lymphocytes), again a trait conserved between mice and humans (Pang et al., 2011; Wahlestedt, Pronk, & Bryder, 2015; Figure 1a).

A recent study using in vivo cell‐tracing labels in mice provided evidence by which this myeloid skewing may occur (Bernitz, Kim, MacArthur, Sieburg, & Moore, 2016). Long‐term HSCs, the most primitive HSC population, are shown to expand numerically with age; however, their frequency within the bone marrow decreases due to accumulation of committed myeloid progenitors (Bernitz et al., 2016). Intriguingly, this study suggests that a division counting mechanism exists within this long‐term HSC compartment, whereby these largely dormant cells undergo four self‐renewal divisions throughout adulthood, and retain memory of their division history, to lose regenerative potential at the fifth division (Bernitz et al., 2016).

### 2.2 Adaptive immune system

#### 2.2.1 Lymphocyte output

The thymus, where T cells develop, begins to involute at puberty in both mice and humans due to age‐related changes that affect both T‐cell progenitors and the thymic microenvironment (Linton & Dorshkind, 2004; Figure 1b). Similarly, decreased hematopoietic tissue in the bone marrow of mice and humans means B‐cell lymphopoiesis also decreases with age (Dorshkind et al., 2009; Ogawa et al., 2000). This reduction in primary lymphoid tissue output means that during adulthood, lymphoid cell regeneration is nearly entirely derived from homeostatic proliferation of the existing T‐ and B‐cell pool, meaning that diversity cannot be increased. Interestingly, a higher ratio of CD4^+^:CD8^+^ T cells in humans has been shown to correlate with frailty and predisposition to disease, but not with “healthy aging” (Strindhall et al., 2007).

#### 2.2.2 B lymphocyte phenotype and function

The aging B‐cell compartment shows a reduced frequency of naïve cells and a commensurate increase in oligoclonal memory B cells. With age, human and mouse B cells produce antibodies with lower affinity for their antigen and have reduced ability to class switch, which hamper immune responses to both vaccination and infection (Kogut, Scholz, Cancro, & Cambier, 2012). Aging also leads to defects in B‐cell tolerance mechanisms which lead to an increased production of autoreactive antibodies and an increased incidence of autoimmune diseases in elderly individuals. These so‐called age‐associated B cells (ABCs), found in both mice and humans and characterized by high expression of T‐bet and CD11c, respond through Toll‐like receptor activation in the absence of BCR stimulation, and can outcompete mature B cells as they have a survival advantage by not being reliant on B‐cell‐activating factor (Naradikian, Hao, & Cancro, 2016).

#### 2.2.3 T lymphocyte phenotype and function

The most well characterized age‐related alterations in the human immune system are in the T‐cell compartment, in particular CD8^+^ T cells, which show reduced numbers in age, commensurate with reduced naïve CD8^+^ T‐cell priming, limiting the ability of CD8^+^ T‐cell responses to be generated toward new infections (Briceno et al., 2016; Figure 1b). In contrast, the CD4^+^ T‐cell compartment is stable with age, although variable between individuals (Wertheimer et al., 2014). Why the CD8^+^ T‐cell compartment shrinks with age, compared to the relatively stable CD4^+^ T‐cell compartment is not clear. CD8^+^ T cells theoretically should receive more signals from the abundantly expressed MHC class I compared to CD4^+^, which receive signals from the MHC class II molecules that are more restricted in expression; therefore, CD8^+^ T cells may homeostatically proliferate at a higher rate than CD4^+^ T cells which may lead to premature senescence. Human T‐cell receptor (TCR) diversity shrinks with age in both CD4^+^ and CD8^+^ compartments, and inequalities in clonal size may compromise immunity or even lead to autoimmunity (Qi et al., 2014). Frequencies of regulatory T cells (Tregs) in humans also alter with age, with Treg numbers increasing (Gregg et al., 2005; Simone, Zicca, & Saverino, 2008), an effect thought to contribute to immune‐suppression in old age (Raynor, Lages, Shehata, Hildeman, & Chougnet, 2012).

Aged CD8^+^ T cells in humans lose expression of co‐stimulatory receptors such as CD28 (Effros, 1997). Accordingly, highly differentiated CD27^‐^CD28^‐^CD45RA^+^CD57^+^ T cells accumulate in older individuals (Czesnikiewicz‐Guzik et al., 2008). These terminally differentiated cells show defective T‐cell receptor (TCR) signaling, decreased proliferative response, exhibit markers of senescence, release pro‐inflammatory cytokines thought to contribute to the “inflamm‐aging” state, and are resistant to apoptosis (reviewed in refs. Michel et al., 2016; Pereira & Akbar, 2016). Aged CD8^+^ T cells also show increased proportions of cells expressing markers usually found on NK cells such as CD16, CD56, CD57, NKp30, KLRG1, CD94, NKG2 family members, and killer‐cell immunoglobulin‐like receptor (KIR) family members (Vallejo, Mueller, et al., 2011). These innate‐like T cells generally exhibit the highly differentiated senescent phenotype and may explain why the senescent CD8^+^ T cells, unlike exhausted T cells, are able to maintain potent effector functions in the absence of a normal proliferative response (Akbar & Henson, 2011; Henson et al., 2014). Why aged CD8^+^, and CD4^+^ T cells to some extent, exhibit NK markers with age is an open question and could be due to chronic antigen‐stimulation, or simply a cell‐intrinsic feature. While innate responses from these αβT cells in the absence of TCR engagement could contribute to autoinflammatory disease (the incidence of which increases with age), evidence suggests that individuals with a higher proportion of innate‐like T cells are less likely to exhibit frailty and immune deterioration (Vallejo, Hamel, et al., 2011).

Another factor thought to contribute to CD8^+^ T‐cell dysfunction is an increase in so‐called virtual memory cells, cells that spontaneously mature with age to express phenotypic memory markers without ever encountering their cognate antigen. These cells express the same markers as those from lymphopenia‐induced homeostatic proliferation, suggesting they develop in response to cytokine stimulation rather than antigen (Haluszczak et al., 2009). Interestingly, recent work using chimeric mice shows that defective CD8^+^ T‐cell function in both naïve and memory compartments is intrinsic to the aged cell, and is not conferred by the aged environment (Quinn et al., 2018). While virtual memory cells have been most clearly observed in mice that are kept in pathogen‐free environments (Nikolich‐Zugich, 2014), the existence of this phenomenon in humans is yet to be conclusively proven. The frequency of memory‐phenotype cells in older humans is certainly higher, and there are certainly T cells specific for antigens never encountered by the host (Su, Kidd, Han, Kotzin, & Davis, 2013). However, these cells may be a result of cross‐reactivity, rather than spontaneous differentiation of naïve cells. In humans, TCR sequencing studies (Qi et al., 2014) and studies of persistent infection (Pulko et al., 2016) suggest that clonal expansions generally maintain a naïve phenotype, but accumulation of these cells may still contribute to the functional defects in proliferation and effector differentiation known to occur in old naïve T cells (Cambier, 2005; Linton & Dorshkind, 2004).

### 2.3 Innate immune system

#### 2.3.1 Monocytes, macrophages, and dendritic cells

The number of circulating blood monocytes does not change with age in healthy humans (Seidler, Zimmermann, Bartneck, Trautwein, & Tacke, 2010). However, the proportion of monocyte subsets appears to shift with an expansion of pro‐inflammatory/non‐classical monocytes (CD16^+^) and a commensurate reduction in classical monocyte (CD14^+^CD16^‐^) frequency in elderly individuals (Seidler et al., 2010). Age‐related decline in both macrophage and dendritic cell number and effector functions have been reported, but a clear delineation of age‐related dysfunction is yet to be defined due to discrepancies in results between studies and groups (Figure 1c). For example, macrophage cytokine release and phagocytosis have both been shown to be reduced, unchanged, or even increased with age (reviewed in refs. Boule & Kovacs, 2017; Shaw, Goldstein, & Montgomery, 2013). Similarly, studies on antigen presentation by dendritic cells have shown mixed results (reviewed in refs. Shaw et al., 2013; Wong & Goldstein, 2013). Differences in species, anatomical location, and stimulation conditions between studies certainly contribute to these discrepancies; nevertheless, this heterogeneity may well reflect the difficulty in isolating the effects of aging from other interfering comorbidities.

#### 2.3.2 Neutrophils

Evidence suggests that circulating neutrophil numbers remain constant with age despite neutrophils exhibiting a shorter half‐life in aged individuals (Fulop et al., 1997; Tortorella et al., 2006). Furthermore, the proliferation of neutrophil precursors in the bone marrow of older people is also reduced (Chatta et al., 1993). In vitro studies have shown neutrophil chemotaxis to be reduced in aged cells. However in vivo*,* the effect of age on neutrophil recruitment is more complex with it reduced in some contexts and increased in others (reviewed in refs. Boule & Kovacs, 2017). Changes in neutrophil function have also been reported in aged individuals, including alternations in cytokine production, reduction in phagocytosis capacity, decreased formation of NETs, and increased production of reactive oxygen species (reviewed in refs. Jackaman et al., 2017; Figure 1c).

#### 2.3.3 NK cells

The number of circulating natural killer (NK) cells is maintained or even increased in old age but NK functional capacity, such as cytotoxicity and cytokine secretion, decreases leaving older individuals vulnerable to tumors and infections (Manser & Uhrberg, 2016; Figure 1c). There is also a skewing of NK cell phenotypes with a decrease in the CD56^hi^ cytokine producing population and an expansion of the CD56^low^ cytotoxic population (Chidrawar, Khan, Chan, Nayak, & Moss, 2006). Interestingly, the aging of the human NK compartment is not recapitulated in mice where NK cells decline with age (Beli et al., 2014; Shehata, Hoebe, & Chougnet, 2015). It is not clear whether this is due to being kept in a pathogen‐free environment, unlike humans, or whether other mechanisms underlie this species difference.

## 3 CHROMATIN CHANGES IN THE AGING IMMUNE SYSTEM

Chromatin, a complex of DNA, RNA, and proteins, is the state in which DNA is packaged within a eukaryotic nucleus. Consisting of repeating nucleosome units (each 147 base pairs of DNA wrapped around an octamer of histone proteins), chromatin structure and epigenetic modifications have critical roles in all aspects of DNA‐related processes including transcription, replication, and repair (Allis and Jenuwein 2016; provide an excellent historical tour of epigenetic knowledge).

Changes in chromatin have been directly linked to the lifespan of model organisms such as yeast, nematodes, and drosophila (Lopez‐Otin et al., 2013). Much work has therefore been done to understand the age‐related epigenetic changes in these model organisms as well as in accelerated aging syndromes (progeria), in order to draw connections to human organismal aging (reviewed in refs. Benayoun, Pollina, & Brunet, 2015; Sen, Shah, Nativio, & Berger, 2016). Indeed, patterns of DNA methylation have been proposed to have utility as a biomarker of aging, a so‐called epigenetic clock (Horvath & Raj, 2018).

**Figure 2:**
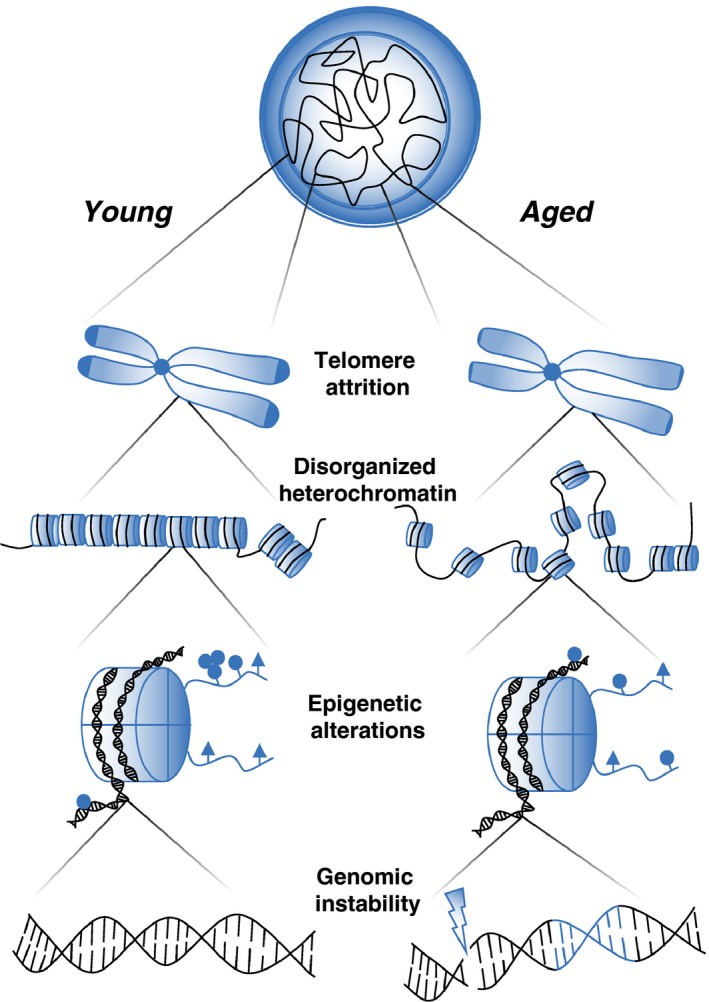
Cellular dysfunction may be caused by the dysregulation of chromatin at many levels. Immune cells from young individuals have a tightly controlled chromatin structure protected from damage by the presence of long telomeres and compacted heterochromatin which associates with the protective nuclear lamina. Chromatin in aged cells exhibits telomere attrition, altered epigenetic marks and genomic instability associated with loss of heterochromatin

Individual cell types show distinct patterns of both epigenetic marks (DNA methylation and histone modifications) and chromatin state (3D genome organization, heterochromatic regions, lamina‐associated domains; Javierre et al., 2016; Lara‐Astiaso et al., 2014; Thurman et al., 2012), all of which affect gene expression and cellular function. It therefore seems likely that different cell types or tissues may be more or less prone to chromatin alterations, and may “age” at different rates. It would therefore seem inappropriate to infer that age‐related changes in one cell type apply ubiquitously. However, relatively few studies have been conducted to examine molecular changes in individual immune cell lineages, perhaps due to difficulties of obtaining sufficient numbers of cells of different lineages such as tissue‐resident lineages or rare cell types (although this is becoming less of an impediment as the sensitivity of technology is improved), perhaps due to the cost associated with sequencing many different cell types, or perhaps simply as it was not deemed necessary when searching for changes well‐characterized in model organisms. We hereafter review the evidence that age‐related changes in immune cell frequencies and function described above are linked to cell‐intrinsic alterations in chromatin modifications or stability (summarized in Figure 2).

### 3.1 Genomic instability in immune cell aging

Maintenance of genomic integrity is mediated by multiple pathways including DNA damage response mechanisms and chromatin remodeling complexes. Over time these pathways lose effectiveness causing age‐related destabilization of the genome (Vijg & Suh, 2013).

#### 3.1.1 DNA damage

DNA damage both promotes cellular senescence in order to allow DNA repair mechanisms and causes activation of the innate immune system in order to clear damaged cells (Soria‐Valles, Lopez‐Soto, Osorio, & Lopez‐Otin, 2017). The reduced capacity of the aged immune system to clear these cells therefore results in an accumulation of genomically damaged and senescent cells within all tissues of the body, including within the immune system itself. Unsurprisingly, defective DNA repair mechanisms lead to premature aging disorders (Hoeijmakers, 2009). However, there is also clear evidence that increased levels of DNA damage are observed in physiologically aged cells, even in murine and human HSCs (Rossi et al., 2007; Rube et al., 2011). There are also age‐related changes in mutation rate, and many mutations that occur within progenitor cells (such as human HSCs) are also carried forward into their differentiated progeny (Welch et al., 2012). Whether individual cell lineages are more or less susceptible to age‐induced DNA damage is unclear. Differential sensitivity of human leukocyte subsets to exogenous mutagens such as H_2_O_2_ and γ‐irradiation has been reported (Mori, Benotmane, Tirone, Hooghe‐Peters, & Desaintes, 2005; Weng, Lu, Weng, & Morimoto, 2008), suggesting that differential effects of age‐induced damage may well contribute to lineage‐specific cellular dysfunction.

#### 3.1.2 Heterochromatin and transposable elements

Higher order chromatin structure can broadly be divided into two major forms, transcriptionally active euchromatin, in which nucleosomes are packed loosely allowing access by transcription factors, and more densely packed heterochromatin which often contains CpG‐methylated DNA and has low transcriptional activity. Loss of repressive heterochromatin has been associated with age‐related dysfunction in a variety of species from yeast to human supporting the global “loss of heterochromatin” theory of aging first proposed over 20 years ago (Tsurumi & Li, 2012; Villeponteau, 1997). This idea that over time heterochromatin domains lose their integrity leading to derepression of silenced genes and aberrant gene activation has been bolstered by recent discoveries of the mobilization of transposable elements from within compacted heterochromatic regions in aging somatic cells in vivo (De Cecco, Criscione, Peterson, et al., 2013), as well as senescent cells in vitro (De Cecco, Criscione, Peckham, et al., 2013). Conversely, extending the lifespan of model organisms has been achieved by genetic manipulation by increasing the levels of repressive heterochromatin and preventing the activation of transposable elements (Wood et al., 2016).

In mammalian immune cells, there is some limited evidence that similar mechanisms may mediate cell‐intrinsic cellular dysfunction. De‐repression of repetitive elements, normally silenced in a heterochromatic state, has been observed in aged mouse HSCs (Sun et al., 2014). Analysis of the chromatin accessibility in human immune cells using the assay for transposase‐accessible chromatin with sequencing (ATAC‐seq; Buenrostro, Giresi, Zaba, Chang, & Greenleaf, 2013) also revealed that advanced age is associated with increased accessibility particularly in quiescent and repressed regions, as opposed to regions critical for cell signaling which show decreased accessibility (Ucar et al., 2017). Interestingly, these findings were most marked in CD8^+^ T cells, a cell type with clear aging‐induced dysfunction (Ucar et al., 2017). These studies support a link with loss of heterochromatin and CD8^+^ T‐cell aging. The role of transposable elements in this context needs to be investigated in future studies.

#### 3.1.3 Nuclear architecture

The nuclear envelope protects the genome from damage and participates in genome maintenance by providing a scaffold for tethering chromatin complexes, particularly transcriptionally silent heterochromatin (Liu et al., 2005). Mutations in genes encoding lamin proteins of the nuclear lamina cause accelerated aging syndromes, and cells from patients with these progeria syndromes predictably show loss of nuclear structure and dissociation of heterochromatin from lamin filaments (Broers, Ramaekers, Bonne, Yaou, & Hutchison, 2006; McCord et al., 2013; Scaffidi, Gordon, & Misteli, 2005; Shumaker et al., 2006). Fibroblasts from aged individual also show nuclear abnormalities and increased DNA damage compared with cells from young individuals (Scaffidi & Misteli, 2006), suggesting that disrupted nuclear structure and chromatin tethering are not an unrelated developmental defect in progeria disorders, but rather a feature of an aged nucleus.

In the immune system, surprisingly little is known of the effects of age on nuclear architecture and chromatin organization in individual immune cell lineages. Murine HSCs show age‐associated downregulation of the *LMNA* gene which encodes both lamin A and its splice‐variant lamin C (Chambers et al., 2007; Rossi et al., 2005), as well as altered DNA repair processes prone to increased mutation rate (Mohrin et al., 2010). Importantly, mature cells of the mouse and human immune systems are notably distinct from cells in other tissues in that few immune cell types express A‐type lamins (Rober, Sauter, Weber, & Osborn, 1990), although differentiation of some dendritic cell and macrophage subsets has been shown to result in acquisition of lamin A/C (Gieseler, Xu, Schlemminger, & Peters, 1993; Prechtel, Turza, Theodoridis, Kummer, & Steinkasserer, 2006; Rober, Gieseler, Peters, Weber, & Osborn, 1990), and T lymphocytes also show transient expression of lamin A/C following activation (Gonzalez‐Granado et al., 2014). Despite the lack of expression of lamin A/C in immune cells themselves, age‐associated down‐regulation of lamin A/C (previously demonstrated in aged cardiomyocytes; Afilalo et al., 2007; and osteoblasts; Duque & Rivas, 2006) may still affect immune cell function, as non‐hematopoietic expression of lamin A/C expression has been demonstrated to be essential for normal T‐ and B‐cell development in chimeric mice (Hale, Frock, Mamman, Fink, & Kennedy, 2010).

Considering the low expression of type A lamins in the immune system, B‐type lamins are considered to be the major lamin filaments in the immune system. There is some evidence from *Drosophila* that lamin B levels reduce with age (Chen, Zheng, & Zheng, 2014), and it is well established in mammalian (non‐immune) cells that levels of both lamin B1 and the lamin B receptor (LBR) are reduced as cells enter senescence. The reduction in lamin B1 in these cells has been demonstrated to lead to irreversible alterations in nuclear architecture including changes in nucleosome density (De Cecco, Criscione, Peckham, et al., 2013), formation of senescence‐associated heterochromatic foci (SAHF) (Sadaie et al., 2013; Shah et al., 2013), and loss of chromatin interactions in regions of heterochromatin (Chandra et al., 2015). It therefore seems highly likely that age‐associated dysregulation of B‐type lamins may have cause functional impairment in the immune system, but this is yet to be directly proven.

### 3.2 Telomere attrition in immune cell aging

Telomeres constitute protective nucleoprotein complexes at the ends of linear chromosomes, which function to ensure complete replication of the genome in cell division, and protect the ends of chromosomes from being recognized as DNA double‐stranded breaks as the ends of telomeres are not blunt ended. Human telomeres terminate in hexameric tandem repeats of TTAGGG which shorten as individuals age or can be extended to render cells immortal through increased expression of the enzyme telomerase, responsible for writing telomeric DNA (Opresko & Shay, 2017).

Mice, which have dysfunctional telomerase rendering telomeres critically short, show premature aging of HSCs (Choudhury et al., 2007). However, it is unclear whether telomeres shorten to this degree over the age of mice and humans to result in this defect in “normal” aging (Martin‐Ruiz, Gussekloo, van Heemst, von Zglinicki, & Westendorp, 2005). Telomere shortening with age has been measured in many human immune cell types including B cells, T cells, granulocytes, and monocytes (Hearps et al., 2012; Rufer et al., 1999; Spyridopoulos et al., 2009). Whether this telomere attrition actually causes the age‐related cellular dysfunction in each of these cell types is however far from clear. Correlations between telomere shortening in CD8^+^ T cell in particular and coronary heart disease (Spyridopoulos et al., 2009) suggest that telomere shortening can indeed cause pathological T‐cell dysfunction. Furthermore, differences in both telomere length and rate of telomere shortening have been observed between different human leukocyte subsets such as CD4^+^ T cells, CD8^+^ T cells, and B cells (Son, Murray, Yanovski, Hodes, & Weng, 2000), as well as between T cells, B cells, and monocytes (Lin et al., 2015), but not between naïve and memory cells (Son et al., 2003), providing a plausible mechanism for the heterogeneity of effects of age in the different cell types within the one individual.

### 3.3 Epigenetic alterations in immune cell aging

Epigenetic control is essential to the healthy development of effector cells of the immune system, as both commitment and differentiation require restriction of alternate fate genes, activation of lineage‐defining transcription factors, and alterations in chromatin accessibility by the transcriptional machinery (reviewed in refs. Busslinger & Tarakhovsky, 2014; Henning, Roychoudhuri, & Restifo, 2018; Schonheit, Leutz, & Rosenbauer, 2015; Shih et al., 2014). A large number of studies have been conducted examining the epigenetic control of each of these processes in immune cell lineages, particularly in T cells. Alterations in any of these processes could feasibly cause the population shifts and functional deficits seen in the aged immune system. For example, failure of epigenetic pathways involved in T‐cell lineage commitment and differentiation (Allan et al., 2012; Hu et al., 2018; Pace et al., 2018) could lead to the myeloid cell skewing seen in aging (Wahlestedt et al., 2015). In contrast, aberrant epigenetic processes involved in formation of memory (Abdelsamed, Zebley, & Youngblood, 2018; Scharer, Bally, Gandham, & Boss, 2017) could lead to the increased proportions of memory cells in aging, whether they be antigen‐experienced or so‐called virtual memory cells (Nikolich‐Zugich, 2014; Su et al., 2013).

We here below review the direct evidence that epigenetic pathways are dysregulated in aged immune cells. Supporting this idea, many studies have shown altered transcriptional profiles in aged immune cell populations (Cao, Gollapudi, Sharman, Jia, & Gupta, 2010; Harries et al., 2011; Mirza, Pollock, Hoelzinger, Dominguez, & Lustgarten, 2011; Reynolds et al., 2015) and alterations in epigenetic regulation are the forefront causative candidate for these changes.

#### 3.3.1 DNA methylation

Change to DNA methylation patterns is probably the best‐studied epigenetic change in aging and has been used to predict the chronological age of human tissues and individuals (Hannum et al., 2013; Horvath, 2013), or perhaps more precisely the “biological age” which is influenced by clinical and environmental parameters to include a measure of health and expected longevity. Predictions have been made from heterogeneous tissues such as lung, liver, and brain tissue as well as whole blood and peripheral blood mononuclear cells, and isolated CD4^+^ T cells, monocytes, and B cells (Horvath, 2013). Remarkably, chronological age can be predicted from just 3 CpG dinucleotide sites in human blood (Weidner et al., 2014). Given the cellular heterogeneity in many of these samples, and the particular changes that occur in blood cell composition with aging, computational methods have been developed to account for age‐related changes in cell proportions (Yuan et al., 2015), but this still does not allow epigenetic changes in specific cell types to be elucidated. Given the differential effects of aging in distinct cell lineages outlined above, this would seem to be essential in order to fully understand the epigenetic mechanisms underlying intrinsic immune cell dysfunction (the difficulties of interpreting epigenome‐wide studies in heterogeneous populations are discussed more fully in ref. Birney, Smith, & Greally, 2016).

DNA methylation levels globally reduce in HSCs and mature leukocytes (and other tissues) as mice or human age (Bjornsson et al., 2008; Fuke et al., 2004; Taiwo et al., 2013). Age‐associated changes in DNA methylation have been reported in a number of human immune cell types including monocytes and CD4^+^ and CD8^+^ T cells (Dozmorov, Coit, Maksimowicz‐McKinnon, & Sawalha, 2017; Reynolds et al., 2014; Tserel et al., 2015; Zhao et al., 2016), and many of these changes appear to be cell type‐specific (Reynolds et al., 2014; Tserel et al., 2015). While DNA methylation at a global level is reduced with age, discrete sites are hypermethylated, particularly sites that are targets of the polycomb repressor complex 2 (PRC2) (Beerman et al., 2013; Horvath et al., 2012; Taiwo et al., 2013), a complex primarily known for imparting the repressive histone mark H3K27me3, but also known to directly cause DNA methylation through the recruitment of DNA methyltransferase (DNMT) enzymes (Vire et al., 2006). Hypermethylation of key genes such as the IL‐7Rα gene and other genes in the IL‐7 signaling pathway has been observed in both human peripheral blood mononuclear cells (PBMCs) and sorted CD8+ T cells (Ucar et al., 2017). This repressive hypermethylation provides a molecular explanation for at least some of the CD8^+^ T‐cell dysfunction seen in old age, as reduced expression of IL‐7Rα in elderly CD8^+^ T cells (Kim, Hong, Dan, & Kang, 2006; Kim, Hwang, Kim, & Kang, 2007) prevents these cells from responding to the critical survival factor IL‐7 (Schluns, Kieper, Jameson, & Lefrancois, 2000).

In CD4^+^ T cells, one study examining human CD28^null^ T cells suggests a link between the loss of the costimulatory molecule CD28 as CD4^+^ T‐cell age, causing alterations in DNA methylation leading to increased expression of inflammasome‐related genes (Suarez‐Alvarez et al., 2017). Another study analyzed publicly available data including datasets from monocytes and CD4^+^ T cells from older people found increased DNA methylation at two sites in the promoter of the *KLF14* gene leading indirectly to suppression of FOXP3 (Johnson et al., 2017). Hypomethylation at the upstream enhancer of *FoxP3* has also been linked to enhanced Treg numbers in older mice (Garg et al., 2014). These isolated reports go some way to linking aberrant DNA methylation with functional deficits in the immune system, but a comprehensive understanding is clearly lacking.

#### 3.3.2 Histone modifications

Post‐translational modifications to the *N*‐terminal tails of histone proteins result in altered chromatin accessibility for the transcriptional machinery, thereby resulting in altered gene expression. Some modifications, such as trimethylation of lysine 9 and lysine 27 of histone‐H3 (H3K9me3 and H3K27me3), are canonical marks of repressed chromatin, whereas others, such as trimethylation of lysine 4 of histone‐H3 (H3K4me3), are associated with open chromatin and active transcription.

Very few studies have been conducted to date examining age‐related genome‐wide changes in histone modifications in mammalian cells, let alone cells of the immune system. One study examining epigenetic changes in old and young mouse HSCs found increased H3K4me3 peaks in the promoters of genes associated with HSC identity and self‐renewal, and conversely increased DNA methylation at transcription factor binding sites in differentiation‐promoting genes (Sun et al., 2014). These changes correlate with changes in expression of these genes (Kowalczyk et al., 2015) and provide a plausible molecular cause for the changes in HSC phenotype and function in old age, described above. Global levels of the constitutive heterochromatin mark H3K9me3, and expression of the prototypical enzyme responsible for depositing this mark, suppressor of variegation 39 homologue 1 (SUV39H1), have also both been shown to reduce with age in a variety of cells types and tissues including mouse and human HSCs (Djeghloul et al., 2016), human stem cells (Zhang et al., 2015), and rat spleen and thymus (Sidler et al., 2013), and therefore may have a causative role in age‐associated immune dysfunction. Supporting this view, Suv39h1 null mice show poor CD8^+^ T‐cell effector responses to *Listeria monocytogenes* infection together with a defect in the silencing of stem‐related and memory‐related genes (Pace et al., 2018). A study using progeria mouse models, however, suggests that Suv39h1 exacerbates premature aging, with deletion of Suv39h1 associated with longer life (Liu et al., 2013). This inconsistency highlights the fact that organismal aging, premature aging syndromes, and age‐associated immune dysfunction may all be driven by different processes, despite all being considered “aging.”

Recent studies examining chromatin accessibility using ATAC‐seq support the idea that the epigenetic landscape of CD8^+^ T cells is altered with age (Moskowitz et al., 2017; Ucar et al., 2017). One study found that naïve and central memory CD8^+^ T cells from older humans showed a loss of chromatin accessibility at gene promoters targeted by the transcription factor NRF1 (Moskowitz et al., 2017), which regulates expression of mitochondrial respiratory chain genes, a plausible although not conclusively proven explanation of the cellular dysfunction seen in the CD8^+^ T‐cell compartment in old age. Another study found that chromatin in human PBMCs from older individuals was more closed at promoters and enhancers associated with T‐cell signaling, such as the IL‐7Rα gene as previously mentioned, compared to that seen in younger individuals (Ucar et al., 2017).

There are a plethora of other chromatin‐modifying proteins which impart and remove a wide variety of histone modifications but which have not been examined in the context of immune aging. A study recently published used a single cell mass cytometry approach to provide the first insight of the effect of aging on 40 of these marks in different human immune cell populations, revealing more variation in chromatin modification profiles in older individuals compared to younger individuals (Cheung et al., 2018). Tracing the chromatin landscape within the same individuals over time will provide a more powerful characterization of the effect of age as this will not be influenced by inter‐individual variation. Candidate histone modifications which are likely to have a role in the mammalian immune system can also be prioritized from studies in other organs and species. For example, regulators of H3K4me3 (Greer et al., 2010) and H3K27me3 (Maures, Greer, Hauswirth, & Brunet, 2011) have been found to control the lifespan of *C. elegans* and may well have a role in immune aging.

Increased levels of H4K20me3 have been found in the liver and kidneys of aged rats (Sarg, Koutzamani, Helliger, Rundquist, & Lindner, 2002). Epigenetic enzymes involved in DNA repair may also play critical roles. For example, SIRT6, an H3K9 deacetylase involved in telomere maintenance (Michishita et al., 2008), is critical for DNA repair and survival of mice past 3 weeks of life (Mostoslavsky et al., 2006). SIRT1 is similarly critical to DNA repair, and SIRT1 target genes have been shown to be dysregulated in the neocortex of the brain of aged mice (Michishita et al., 2008). The histone deacetylases HDAC1 and HDAC2 also have roles in DNA repair (Miller et al., 2010) and have been linked to cellular senescence and aging in mouse brain and liver, and human fibroblasts (Chouliaras et al., 2013; Willis‐Martinez, Richards, Timchenko, & Medrano, 2010). Age‐associated loss of histones and altered nucleosome occupancy has been seen in cultured human fibroblast cells as they are serially passaged (O'Sullivan, Kubicek, Schreiber, & Karlseder, 2010), adding a further layer of complexity to histone‐based regulation. Further work is clearly needed to characterize global age‐relating alterations in histone modifications in immune cell types.

Dysregulation of histone post‐translational modifications does not need to occur genomewide to have marked influences on cellular function as changes in the epigenetic regulation of key genes can lead to cellular senescence and aging. For example, decreased histone acetylation at the mouse *Bach2* locus has been linked to CD4^+^ T‐cell senescence (Kuwahara et al., 2014). Perhaps the best‐characterized example of this in the immune system is the epigenetic regulation of the INK4 family of cyclin‐dependent kinase (CDK) inhibitors encoded by the *INK4A‐ARF* locus, which leads to cellular senescence and aging of a variety of cell types including mouse HSCs (Janzen et al., 2006) and mouse and human fibroblasts (Bracken et al., 2007). This locus is epigenetically silenced in young cells by polycomb repressive complexes PRC1 and PRC2 which impart the silencing H3K27me3 mark (Bracken et al., 2007). As cells age, enhancer of zeste homologue 2 (EZH2), the enzymatic component of PRC2, is down‐regulated (Kamminga et al., 2006; Sun et al., 2014). Additionally, H3K27me3 is demethylated by Jumonji D3 (Agger et al., 2009; Barradas et al., 2009), and the activating mark H3K4me3 is imparted by mixed‐lineage leukemia 1 (MLL1) (Kotake, Zeng, & Xiong, 2009), together resulting in de‐repression of the INK4 family of cyclin‐dependent kinase (CDK) inhibitors leading to cellular senescence. Histone acetylation by MYST family members at this locus also regulates the senescence of HSCs and other cell types (Perez‐Campo et al., 2014; Sheikh, Phipson, et al., 2015). However, the relevance of this mechanism to age‐related dysfunction is unknown, with most work focusing on harnessing this pathway in the treatment of blood cancers (Baell et al., 2018; Sheikh, Lee, et al., 2015).

Complex interplay between the ARF protein encoded by the *INK4A‐ARF* locus, the p53 tumor suppressor, and the histone demethylase Jumonji D3 may also occur as this has been reported in mouse neural stem cells (Sola et al., 2011), HEK293 cells, and glioblastoma cells (Ene et al., 2012). In these cell types, Jumonji D3 demethylation of the *INK4A‐ARF* locus leads to expression of the ARF protein which causes nuclear accumulation of p53 and increased transcription of the *p21* gene, a cyclin‐dependent kinase inhibitor which induces cellular senescence (Ene et al., 2012). Whether this occurs in immune cell types is yet to be shown. Expression of p16^INK4A^, another protein encoded in the *INK4A‐ARF* locus, has been proposed to have utility as a biomarker of aging when measured in human peripheral T lymphocytes (Y. Liu et al., 2009), supporting a role for this gene in differentiated immune cells, not just stem cells.

## 4 DETERMINING MOLECULAR CAUSALITY IN IMMUNE DYSFUNCTION

**Figure 3:**
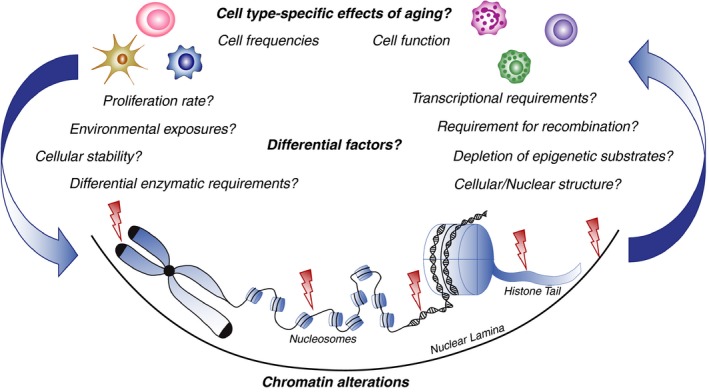
Differential susceptibility of cell types to age‐related dysfunction may be due to intrinsic or extrinsic factors. Chromatin alterations may drive cellular dysfunction, or cellular dysfunction may result in chromatin alterations. Regardless, understanding connections between chromatin state and cellular dysfunction may enable manipulation of chromatin‐modifying pathways to restore a youthful cellular phenotype and protective immunity

As summarized above, there is no doubt that aging is both associated with alterations in immune cell frequency and function, as well as a suite of chromatin changes in immune and non‐immune cell types. Determining causality between these two phenomena is somewhat more difficult. Chromatin alterations observed with age may drive the cellular dysfunction, or alternatively, cellular dysfunction may cause chromatin alterations, or perhaps both (see Figure 3). Different cell types are clearly differentially susceptible to age‐related changes which may reflect intrinsic differences in chromatin requirements, such as the initiation or maintenance of transcriptional programs, or requirements for epigenetic enzymes to alter chromatin state in processes such as differentiation. Some cell types have different requirements for 3‐dimensional genome organization such as that required to perform V(D)J recombination in the adaptive but not the innate immune system (Rivera‐Munoz et al., 2007; Shih & Krangel, 2013). Alternatively, differences in cellular environment such as exposure to DNA damage‐inducing stimuli could influence the chromatin environment (Chen, Hales, & Ozanne, 2007). Similarly, cell types with high rates of turnover or less protective nuclear lamina may result in accumulation of mutations and disruption in chromatin organization (Criscione, Teo, & Neretti, 2016).

Regardless of whether chromatin alterations or cellular phenotype or environment are considered the “cause” of the differential effects of aging, if the phenotype can be rescued through modulating chromatin‐modifying enzymes then the chromatin alteration can surely be viewed as the molecular driver of age‐related immune dysfunction. This is clearly the case when telomerase activity is restored (Bodnar et al., 1998), and there is some evidence that ectopic Ezh2 expression can partially prevent cellular senescence (Ito, Teo, Evans, Neretti, & Sedivy, 2018)), albeit not yet shown in immune cell types. More work needs to be done in this space to truly confirm a causal role for each of the chromatin alterations discussed above, with the rescue of immunity the ultimate goal in this context, rather than increased lifespan per se.

## 5 CONCLUDING REMARKS

The quest for a central unified theory of aging has revealed several molecular hallmarks common across cell type and species. We can infer a lot from studies in model organisms; however, direct evidence demonstrating the molecular basis of cell type‐specific dysfunction within the human immune system remains scarce. Part of the reason for this may be the technical limitations of performing genomewide epigenomic analysis on limited starting material, such as that required for human samples and smaller cellular populations from mice and other non‐human mammals. Rapid technological development including the advent and more wide‐spread use of single cell RNA‐seq and ATAC‐seq methodologies, and advances in ChIP‐seq‐based assays, mean molecular analyses of small cell numbers is now more achievable than ever. Applying these technological advances to the phenotypic changes observed in the aged immune system will rapidly advance this field and hopefully reveal key molecular events which underpin the loss of immune function in old age. Through this approach and the burgeoning field of epigenetic drug discovery (Tough, Tak, Tarakhovsky, & Prinjha, 2016), it is hoped that youthful immunity can be restored in old age.

## CONFLICT OF INTEREST

The authors declare that the research was conducted in the absence of any commercial or financial relationships that could be construed as a potential conflict of interest.

## AUTHOR CONTRIBUTIONS

Both authors have made substantial, direct, and intellectual contribution to the work and approved it for publication.
